# Preoperative Predictors of Insufficient Future Liver Remnant Hypertrophy Following Percutaneous Transhepatic Portal Vein Embolization: A Single-Center Retrospective Cohort Study

**DOI:** 10.7759/cureus.110946

**Published:** 2026-06-16

**Authors:** Masato Saito, Kenichiro Nitta, Hiroki Sato, Akemi Ohtani, Hiroki Okuda, Tomokazu Hasegawa, Masanori Someya

**Affiliations:** 1 Division of Radiation Oncology and Interventional Radiology, Department of Radiology, Sapporo Medical University, Sapporo, JPN

**Keywords:** future liver remnant, hepatic regeneration, liver hypertrophy, liver volumetry, portal vein embolization, preoperative predictor, rapid turnover protein, transthyretin

## Abstract

Background

Percutaneous transhepatic portal vein embolization (PTPE) is a widely used preoperative procedure to augment future liver remnant (FLR) volume before major hepatectomy. However, a subset of patients fails to achieve sufficient FLR hypertrophy following PTPE. Early identification of patients at risk for insufficient hypertrophy would facilitate the timely planning of additional strategies. This study aimed to identify preoperative predictors of insufficient FLR hypertrophy following PTPE, with particular focus on nutritional markers.

Methods

We retrospectively reviewed 87 patients who underwent PTPE at our institution between April 2010 and February 2026. After excluding 18 patients who underwent left-sided PTPE and eight with confirmed recanalization, 61 patients were included in the final analysis. Insufficient FLR hypertrophy was defined as a hypertrophy ratio (post-PTPE FLR volume/pre-PTPE FLR volume) < 1.3. Univariable and multivariable logistic regression analyses were performed to identify preoperative predictors. Two patients were excluded from the multivariable analysis and subsequent analyses due to missing transthyretin (TTR) values, resulting in an analytical sample of 59 patients for these analyses. Receiver operating characteristic (ROC) curve analysis was performed for independent predictors, and patients were stratified into four risk groups based on the identified cutoff values.

Results

Insufficient FLR hypertrophy was observed in 24 patients (39.3%). On univariable analysis, pre-PTPE FLR volume (p = 0.001), pre-PTPE FLR ratio (p = 0.005), total liver volume (TLV) (p = 0.022), total bilirubin (TB) (p = 0.046), cholinesterase (CHE) (p = 0.028), TTR (p = 0.047), and HH15 (p = 0.027) were significantly associated with insufficient hypertrophy. Multivariable logistic regression identified pre-PTPE FLR volume (odds ratio (OR) = 4.277, 95% confidence interval (CI): 1.873-9.767; p = 0.001) and TTR (OR = 0.874, 95% CI: 0.770-0.993; p = 0.039) as independent predictors. ROC analysis yielded an area under the curve (AUC) of 0.809 for pre-PTPE FLR volume (optimal cutoff: 428.3 mL), and 0.660 for TTR (optimal cutoff: 14.9 mg/dL); the combined model incorporating both predictors yielded an AUC of 0.847 (95% CI: 0.740-0.936). When patients were stratified by these cutoff values, all patients in the high-risk group (pre-PTPE FLR volume ≥ 428.3 mL and TTR ≤ 14.9 mg/dL; n = 7) experienced insufficient hypertrophy, compared with only 13.3% in the low-risk group (neither risk factor present; n = 30).

Conclusions

Pre-PTPE FLR volume was identified as the strongest preoperative predictor of insufficient FLR hypertrophy following PTPE, with TTR demonstrating a significant independent association as a complementary predictor. The combination of these two variables, obtainable from routine computed tomography (CT) volumetry and laboratory testing, may inform preoperative risk stratification prior to PTPE, although the cutoff values were derived exploratorily and require external validation in larger, multicenter cohorts.

## Introduction

Surgical resection remains the only curative treatment for hepatobiliary malignancies, including hepatocellular carcinoma, cholangiocarcinoma, and gallbladder cancer; however, major hepatectomy is frequently required to achieve curative resection. Insufficient future liver remnant (FLR) volume is a well-recognized major cause of post-hepatectomy liver failure (PHLF) [1,2], making adequate preoperative FLR augmentation critically important.

Percutaneous transhepatic portal vein embolization (PTPE) is an established preoperative procedure that selectively occludes portal flow to the hepatic lobe scheduled for resection, thereby inducing compensatory hypertrophy of the non-embolized lobe and increasing FLR volume [3-5]. Nevertheless, approximately 15-20% of patients who undergo PTPE fail to proceed to resection due to either disease progression or insufficient FLR hypertrophy [3,4,6]. Although disease progression is often difficult to control, insufficient FLR hypertrophy represents a potentially addressable problem through preoperative intervention.

Previous studies have reported various preoperative predictors for insufficient FLR hypertrophy after PTPE, encompassing clinical and technical factors [7], volumetric and functional indicators such as FLR ratio and indocyanine green (ICG) clearance metrics [8,9], the impact of prior chemotherapy [10,11], and vascular and radiological cofactors [12,13]. However, results across these studies are difficult to compare directly, as the metrics used to quantify the hypertrophic response also vary, including hypertrophy ratio (post-PTPE FLR volume/pre-PTPE FLR volume) [10,14] and degree of hypertrophy (DH; the difference in standardized FLR before and after PTPE) [15].

If patients at risk of insufficient hypertrophy could be identified preoperatively, early planning of additional strategies would become feasible, potentially reducing unnecessary waiting periods and minimizing the risk of tumor progression during the augmentation phase.

The purpose of this study was to identify preoperative predictors of insufficient FLR hypertrophy following PTPE, with particular focus on nutritional markers.

## Materials and methods

Study design and setting

This was a single-center retrospective cohort study conducted at Sapporo Medical University, Sapporo, Japan, to identify preoperative predictors of insufficient FLR hypertrophy following PTPE.

Ethical approval

This study was approved by the Institutional Review Board (IRB) of Sapporo Medical University (approval number: 372-297). As this was a retrospective study, the requirement for written informed consent was waived; instead, an opt-out consent procedure was employed in accordance with institutional guidelines. Clinical data were anonymized prior to analysis.

Patient selection

**Figure 1:**
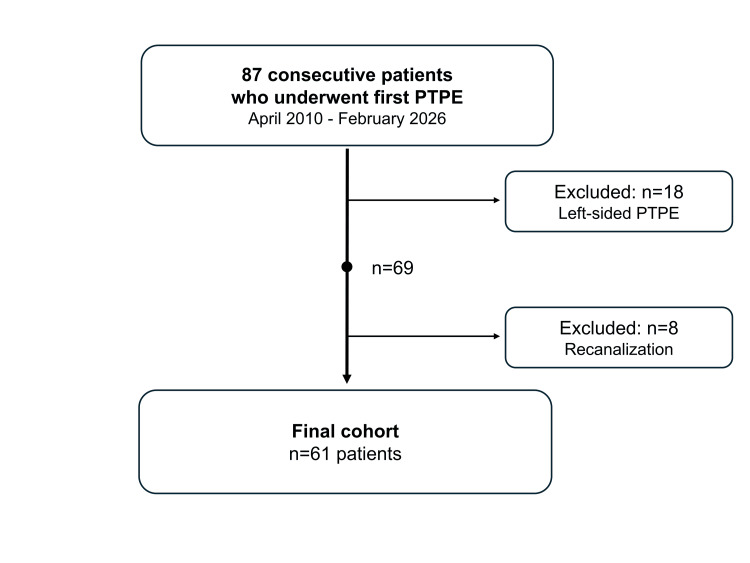
Flowchart of patient selection Of 87 consecutive patients who underwent their first PTPE between April 2010 and February 2026, 18 were excluded due to left-sided PTPE performed in anticipation of extended left hepatectomy or left trisectionectomy, and eight were excluded due to confirmed recanalization on post-PTPE imaging. A total of 61 patients were included in the final analysis. Of these, two patients were excluded from analyses requiring TTR values (multivariable logistic regression, ROC analysis for TTR, risk stratification, and multivariable linear regression in the sensitivity analysis) due to missing preoperative TTR data, resulting in an analytic sample of 59 patients for these analyses. Abbreviations: PTPE: percutaneous transhepatic portal vein embolization; TTR: transthyretin; ROC: receiver operating characteristic.

We retrospectively reviewed 87 consecutive patients who underwent PTPE at our institution between April 2010 and February 2026. Patients were excluded if they met any of the following criteria: (1) left-sided PTPE performed in anticipation of extended left hepatectomy or left trisectionectomy (n = 18); or (2) confirmed recanalization on post-PTPE imaging (n = 8). Only patients undergoing their first PTPE were included. A total of 61 patients were included in the final analysis (Figure 1).

Indications for PTPE were determined at multidisciplinary team (MDT) conferences. Preoperative FLR volume was assessed using a previously reported liver function scoring system [16], which comprises four variables: ICG-R15, serum antithrombin III (AT-III), and two technetium-99m galactosyl human serum albumin (⁹⁹ᵐTc-GSA) hepatic scintigraphy indices (HH15 and LHL15). Each variable is awarded 1 to 4 points, with a total score range of 4-16 points. Based on linear regression analysis, each 1-point increase in the score permits an approximately 5% greater reduction in total liver volume (TLV), allowing safe resection of up to 65% of TLV in patients achieving the maximum score of 16. PTPE was indicated when the pre-PTPE FLR volume fell below the patient-specific minimum threshold defined by this scoring system.

PTPE procedure

All PTPE procedures were performed by interventional radiologists experienced in percutaneous transhepatic techniques. Under ultrasound guidance, a 5 Fr sheath was placed into the portal vein via an ipsilateral approach in all cases. A 4 Fr hook-shaped catheter and a 2.0 Fr or 2.4 Fr microcatheter were used in a coaxial technique. The microcatheter was selectively advanced to the level of portal subsegmental branches, and embolization was performed sequentially in each branch. The embolic agent used in all cases (n = 61) was cut gelatin sponge. In three cases, metallic coils were additionally deployed into the target portal branches due to residual portal flow following gelatin sponge embolization.

Imaging assessment and liver volumetry

Contrast-enhanced computed tomography (CT) was performed before PTPE and three to four weeks after the procedure, and liver volumetry was performed at both time points using Ziostation2 (Fujifilm Healthcare, Tokyo, Japan). All volumetric measurements were performed by a single experienced radiological technologist using a standardized volumetric protocol throughout the study period. Formal intra-observer variability assessment was not performed. FLR volume and TLV were calculated by three-dimensional volumetry using portal venous phase images from contrast-enhanced CT. FLR volume (mL) before and after PTPE was measured by tracing the contour of the planned liver remnant (the non-embolized lobe). TLV was defined as the directly measured volume (mL) obtained by tracing the contour of the entire liver on the same CT images. Vascular structures, including the portal vein, hepatic veins, and inferior vena cava, as well as benign hepatic lesions such as hepatic cysts and hemangiomas, were excluded from all volumetric measurements.

The following parameters were calculated: pre-PTPE FLR volume (mL); post-PTPE FLR volume (mL); TLV (mL); pre-PTPE FLR ratio (%), calculated as $\frac{\text{pre-PTPE FLR volume (mL)}}{\text{pre-PTPE TLV (mL)}} \times 100$; and hypertrophy ratio, calculated as $\frac{\text{post-PTPE FLR volume (mL)}}{\text{pre-PTPE FLR volume (mL)}}$.

Outcome definition

The primary outcome was insufficient FLR hypertrophy, defined as a hypertrophy ratio ($\frac{\text{post-PTPE FLR volume (mL)}}{\text{pre-PTPE FLR volume (mL)}}$) < 1.3. This cutoff was based on previously published reports indicating a median hypertrophy ratio of approximately 1.29 in similar cohorts [10,14].

Preoperative variables

Preoperative clinical and laboratory data collected within seven days before PTPE were evaluated as candidate predictors.

The following variables were collected: (i) Patient characteristics and clinical information: age, diabetes mellitus, viral hepatitis, history of preoperative chemotherapy, and use of antithrombotic agents. (ii) Laboratory values: serum aspartate aminotransferase (AST), alanine aminotransferase (ALT), AST/ALT ratio, total bilirubin (TB), alkaline phosphatase (ALP), platelet count (Plt), albumin (Alb), transthyretin (TTR), cholinesterase (CHE), prothrombin time (PT%), AT-III, and the Controlling Nutritional Status (CONUT) score. The CONUT score was calculated from serum albumin, total cholesterol, and total lymphocyte count, as previously described [17]. (iii) Hepatic functional reserve indices: ICG retention rate at 15 minutes (ICG-R15) and hepatic scintigraphy indices (HH15 and LHL15) based on technetium-99m-labeled galactosyl human serum albumin (⁹⁹ᵐTc-GSA) scintigraphy. (iv) Liver volumetry parameters: TLV (mL), pre-PTPE FLR volume (mL), and pre-PTPE FLR ratio (%).

Sensitivity analysis

To address the potential mathematical dependency between pre-PTPE FLR volume and the hypertrophy ratio, a sensitivity analysis was performed using the standardized FLR volume increase (ΔsFLR, %), calculated as $\frac{\text{absolute FLR volume increase}}{\text{estimated standard liver volume (SLV)}} \times 100$. SLV was estimated using the Vauthey formula: $\text{SLV (mL)} = 794.41 + 1267.28 \times \text{body surface area (m}^2\mathrm{)}$, where body surface area was calculated using the Mosteller formula.

To assess whether the association between TTR and insufficient FLR hypertrophy was independent of systemic inflammation, two additional multivariable logistic regression models were constructed by adding C-reactive protein (CRP) and neutrophil-to-lymphocyte ratio (NLR) separately as covariates to the primary multivariable model.

Statistical analysis

Continuous variables are presented as median (interquartile range (IQR)), and categorical variables are presented as count (%).

Univariable logistic regression analysis was performed to assess the association between each candidate predictor and the primary outcome (insufficient FLR hypertrophy). Odds ratios (OR) and 95% confidence intervals (CI) were calculated. Variables with p < 0.05 on univariable analysis were eligible for inclusion in the multivariable model. The number of variables entered into the multivariable logistic regression model was limited to two, considering the events per variable (EPV) relative to the number of events (n = 24). Pre-PTPE FLR volume was selected as the representative volumetric variable given its strongest univariable association; TTR was selected over CHE (Spearman’s ρ = 0.558) given its shorter half-life and greater sensitivity to acute changes in hepatic synthetic function. Pre-PTPE FLR volume and TTR were entered into the multivariable model using the enter method. Two patients were excluded from the multivariable model and subsequent analyses due to missing TTR values, resulting in an analytic sample of 59 patients for the multivariable logistic regression, receiver operating characteristic (ROC) analysis for TTR, and risk stratification. Model fit was assessed using Nagelkerke R² and the Hosmer-Lemeshow test.

As an exploratory analysis, ROC curves were constructed for pre-PTPE FLR volume and TTR. The area under the curve (AUC) and 95% CI were calculated using the bootstrap method (n = 2,000 iterations), and optimal cutoffs were determined by the Youden index. A combined ROC curve incorporating both variables was also constructed using predicted probabilities from the multivariable model. Internal validation was performed using leave-one-out cross-validation (LOOCV), in which each patient was iteratively excluded and the model was refitted on the remaining patients to generate a predicted probability; the AUC was then calculated across all held-out predictions.

Patients (n = 59) were then stratified into four groups based on these cutoffs, and hypertrophy ratios were compared using the Kruskal-Wallis test with Bonferroni-corrected post-hoc Mann-Whitney U tests.

For the sensitivity analysis, Spearman rank correlation and multivariable linear regression analyses (covariates: pre-PTPE FLR volume and TTR) were performed using ΔsFLR as the outcome measure.

All statistical analyses were performed using Python (version 3.14.4; Python Software Foundation, Wilmington, DE, USA) with the pandas, scipy, statsmodels, and scikit-learn libraries. Statistical significance was defined as two-sided p < 0.05.

## Results

Patient characteristics

A total of 61 patients were included in the final analysis (Table 1). The median age was 70 years (IQR: 65-74 years). Diabetes mellitus was present in 17 patients (27.9%) and viral hepatitis in 24 patients (39.3%); no patient had underlying liver cirrhosis. Twelve patients (19.7%) had a history of preoperative chemotherapy. The planned surgical procedure was hepatectomy alone in 47 patients (77.0%) and combined hepatectomy with pancreaticoduodenectomy (PD) in 14 patients (23.0%). The cohort comprised predominantly biliary tract malignancies; perihilar cholangiocarcinoma was the most common diagnosis (n = 32, 52.5%), followed by gallbladder cancer, including cystic duct cancer (n = 14, 23.0%), intrahepatic cholangiocarcinoma (n = 7, 11.5%), and distal cholangiocarcinoma (n = 6, 9.8%). No patient had hepatocellular carcinoma.

**Table 1:** Patient characteristics (n = 61) Continuous variables are presented as median (interquartile range (IQR)). Categorical variables are presented as n (%). † Viral hepatitis includes both active infection (HBs antigen-positive; n = 4) and past infection (anti-HBc antibody-positive; n = 20). All 24 patients had HBV infection; no patient had HCV infection. Abbreviations: IQR, interquartile range; HBV, hepatitis B virus; HCV, hepatitis C virus; AST, aspartate aminotransferase; ALT, alanine aminotransferase; ALP, alkaline phosphatase (IFCC method); PD, pancreaticoduodenectomy; TTR, transthyretin (formerly known as prealbumin); CONUT, Controlling Nutritional Status; ICG-R15, indocyanine green retention rate at 15 minutes; HH15 and LHL15, ⁹⁹ᵐTc-GSA scintigraphic hepatic function indices.

Variable	All patients (n = 61)
Demographics and Comorbidities	
Age (years), median (IQR)	70 (65 - 74)
Diabetes mellitus - n (%)	17 (27.9)
Viral hepatitis^†^ - n (%)	24 (39.3)
HBV	24 (39.3)
HCV	0 (0)
Liver cirrhosis - n (%)	0 (0)
Preoperative chemotherapy - n (%)	12 (19.7)
Antithrombotic therapy - n (%)	11 (18.0)
Primary Diagnosis	
Perihilar cholangiocarcinoma - n (%)	32 (52.5)
Gallbladder cancer (including cystic duct cancer) - n (%)	14 (23.0)
Intrahepatic cholangiocarcinoma - n (%)	7 (11.5)
Distal cholangiocarcinoma - n (%)	6 (9.8)
Metastatic liver cancer - n (%)	1 (1.6)
Hepatic echinococcosis - n (%)	1 (1.6)
Planned Surgery	
Hepatectomy alone - n (%)	47 (77.0)
Extended right hepatectomy	39 (63.9)
Right trisectionectomy	5 (8.2)
Right hepatectomy	3 (4.9)
Hepatectomy + pancreaticoduodenectomy - n (%)	14 (23.0)
Extended right hepatectomy + PD	13 (21.3)
Right trisectionectomy + PD	1 (1.6)
Embolic Agent	
Gelatin sponge alone - n (%)	58 (95.1)
Gelatin sponge + coil - n (%)	3 (4.9)
Preoperative Laboratory Values, median (IQR)	
Total bilirubin (mg/dL)	0.7 (0.6 - 1.0)
AST (U/L)	28.5 (23.0 - 44.3)
ALT (U/L)	35.0 (24.0 - 60.3)
AST/ALT ratio	0.85 (0.68 - 1.08)
ALP (U/L)	328 (207 - 522)
Albumin (g/dL)	3.7 (3.4 - 4.0)
Prothrombin time (%)	101.1 (89.0 - 107.2)
Antithrombin III (%)	104.5 (93.0 - 113.8)
Platelets (×10³/μL)	20.9 (17.4 - 27.0)
Transthyretin (TTR) (mg/dL)	20.9 (16.7 - 24.6)
Cholinesterase (U/L)	260 (209 - 305)
CONUT score	2 (1 - 3)
ICG-R15 (%)	8.0 (5.0 - 11.5)
Hepatic Scintigraphy, median (IQR)	
HH15	0.570 (0.540 - 0.620)
LHL15	0.950 (0.940 - 0.960)

The median pre-PTPE FLR volume was 401.0 mL (IQR: 327.3-496.3 mL), and the median pre-PTPE FLR ratio was 35.7% (IQR: 30.0-39.6%). The median pre-PTPE TLV was 1,153.1 mL (IQR: 1,026.8-1,342.4 mL). The median TTR was 20.9 mg/dL (IQR: 16.7-24.6 mg/dL), and the median ICG-R15 was 8.0% (IQR: 5.0-11.5%). The median CONUT score was 2 (IQR: 1-3).

Changes in FLR volume after PTPE

After PTPE, FLR volume increased (hypertrophy ratio > 1.0) in 60 of 61 patients. In one patient, the hypertrophy ratio was below 1.0 (minimum value: 0.978), indicating no FLR growth. The median post-PTPE FLR volume was 550.4 mL (IQR: 454.5-636.0 mL), and the median post-PTPE FLR ratio was 47.6% (IQR: 41.1-51.4%). The median hypertrophy ratio was 1.387 (IQR: 1.224-1.539) (Table 2). For reference, the median hypertrophy ratio, including the eight excluded recanalization cases (n = 69), was 1.363 (IQR: 1.217-1.516).

**Table 2:** Liver volume profile before and after PTPE (n = 61) Continuous variables are presented as median (interquartile range (IQR)). Hypertrophy ratio = post-PTPE FLR volume/pre-PTPE FLR volume. Abbreviations: PTPE, percutaneous transhepatic portal vein embolization; IQR, interquartile range; TLV, total liver volume; FLR, future liver remnant.

Variable	Pre-PTPE	Post-PTPE
Liver Volume Parameters, median (IQR)		
Total liver volume, TLV (mL)	1153.1 (1026.8 - 1342.4)	1168.8 (1054.8 - 1367.1)
FLR volume (mL)	401.0 (327.3 - 496.3)	550.4 (454.5 - 636.0)
FLR ratio (%)	35.7 (30.0 - 39.6)	47.6 (41.1 - 51.4)
Hypertrophy ratio	-	1.387 (1.224 - 1.539)

Insufficient hypertrophy, defined as a hypertrophy ratio < 1.3, was observed in 24 patients (39.3%), while 37 patients (60.7%) achieved sufficient hypertrophy (≥ 1.3). The median hypertrophy ratio was 1.178 (IQR: 1.055-1.251) in the insufficient hypertrophy group and 1.488 (IQR: 1.409-1.666) in the sufficient hypertrophy group. The median interval from PTPE to follow-up CT was 22.0 days in both the sufficient and insufficient hypertrophy groups (Mann-Whitney U test, p = 0.207).

Clinical outcomes

Of the 61 patients who underwent PTPE, 47 (77.0%) proceeded to surgery. The remaining 14 patients (23.0%) did not undergo surgery due to tumor progression (n = 7) or insufficient FLR volume (n = 7). No patient developed postoperative liver failure as defined by the ISGLS (International Study Group of Liver Surgery) criteria (bilirubin > 3 mg/dL on or after postoperative day 5), among those who underwent surgery.

Univariable analysis

Univariable logistic regression identified the following variables as significantly associated with insufficient hypertrophy: pre-PTPE FLR volume (OR = 3.265, 95% CI: 1.661-6.417, per 100mL increase; p = 0.001), pre-PTPE FLR ratio (OR = 1.161, 95% CI: 1.047-1.287; p = 0.005), TLV (OR = 1.003, 95% CI: 1.000-1.005; p = 0.022), total bilirubin (OR = 2.646, 95% CI: 1.020-6.865; p = 0.046), CHE (OR = 0.991, 95% CI: 0.983-0.999; p = 0.028), TTR (OR = 0.901, 95% CI: 0.812-0.999; p = 0.047), and HH15 (OR = 1.108, 95% CI: 1.012-1.214; p = 0.027) (Table 3). AT-III showed a trend toward significance (OR = 0.971, 95% CI: 0.938-1.004; p = 0.083) but did not reach the threshold for statistical significance. Age, diabetes mellitus, viral hepatitis, preoperative chemotherapy, antithrombotic therapy, AST, ALT, AST/ALT ratio, ALP, albumin, PT%, platelets, CONUT score, ICG-R15, and LHL15 were not significantly associated with insufficient hypertrophy.

**Table 3:** Univariable logistic regression analysis Outcome: insufficient hypertrophy (hypertrophy ratio < 1.3). Continuous variables are presented as median (interquartile range). Categorical variables are presented as n (%). An asterisk (*) indicates statistical significance (p < 0.05). n = 37 (sufficient hypertrophy) and n = 24 (insufficient hypertrophy) for all variables except TTR; n = 36 and n = 23, respectively, for TTR due to missing preoperative values in two patients. Odds ratios (ORs) are presented per the following increment units: pre-PTPE FLR volume and TLV, per 100 mL; AST, ALT, and cholinesterase, per 10 U/L; ALP, per 100 U/L; prothrombin time and antithrombin III, per 10%; platelets, per 10 × 10³/μL; HH15 and LHL15, per 0.01 unit (rescaled ×100 prior to analysis); all other continuous variables, per 1 unit. Abbreviations: OR, odds ratio; CI, confidence interval; AST, aspartate aminotransferase; ALT, alanine aminotransferase; ALP, alkaline phosphatase (IFCC method); TTR, transthyretin; CONUT, Controlling Nutritional Status; ICG-R15, indocyanine green retention rate at 15 minutes; HH15 and LHL15, ⁹⁹ᵐTc-GSA scintigraphic hepatic function indices; TLV, total liver volume; FLR, future liver remnant; PTPE, percutaneous transhepatic portal vein embolization.

Variable	Sufficient hypertrophy	Insufficient hypertrophy	OR	95% CI	p-value
Demographics and Comorbidities					
Age (years)	70.0 (65.0-74.0)	71.5 (64.8-74.5)	1.021	0.961-1.084	0.501
Diabetes mellitus	9 (24.3)	8 (33.3)	1.556	0.501-4.831	0.445
Viral hepatitis	17 (45.9)	7 (29.2)	0.484	0.163-1.444	0.193
Preoperative chemotherapy	6 (16.7)	6 (26.1)	1.765	0.491-6.337	0.384
Antithrombotic therapy	8 (21.6)	3 (12.5)	0.518	0.123-2.168	0.371
Preoperative Laboratory Values					
Total bilirubin (mg/dL)	0.6 (0.5-0.9)	1.0 (0.6-1.4)	2.646	1.020-6.865	0.046*
AST (U/L)	27.0 (23.0-43.0)	29.0 (23.0-46.0)	0.999	0.763-1.309	0.996
ALT (U/L)	35.0 (24.0-62.0)	30.0 (21.0-57.0)	0.962	0.811-1.141	0.654
AST/ALT ratio	0.8 (0.7-1.0)	0.9 (0.8-1.2)	2.295	0.633-8.321	0.206
ALP (U/L)	329.0 (226.0-499.0)	327.0 (153.5-619.5)	1.052	0.922-1.199	0.453
Albumin (g/dL)	3.7 (3.4-4.1)	3.7 (3.3-4.0)	0.573	0.165-1.990	0.381
Prothrombin time (%)	102.1 (91.0-109.0)	97.0 (87.5-105.5)	0.863	0.598-1.246	0.432
Antithrombin III (%)	106.0 (97.0-118.0)	99.0 (90.0-107.5)	0.742	0.529-1.040	0.083
Platelets (×10³/μL)	21.4 (17.4-30.1)	19.1 (17.0-22.6)	0.973	0.823-1.150	0.746
Transthyretin (TTR) (mg/dL)	22.1 (17.5-25.0)	17.5 (14.0-22.9)	0.901	0.812-0.999	0.047*
Cholinesterase (U/L)	286.0 (240.0-323.0)	218.0 (176.0-274.0)	0.915	0.846-0.990	0.028*
CONUT score	2.0 (1.0-3.0)	3.0 (1.5-3.0)	1.175	0.820-1.683	0.380
ICG-R15 (%)	7.3 (4.9-10.6)	9.2 (5.8-12.4)	1.052	0.974-1.137	0.196
Hepatic Scintigraphy					
HH15	0.570 (0.540-0.620)	0.620 (0.570-0.670)	1.108	1.012-1.214	0.027*
LHL15	0.951 (0.936-0.961)	0.950 (0.935-0.954)	0.897	0.706-1.140	0.375
Liver Volume Parameters					
Total liver volume, TLV (mL)	1094.3 (983.2-1291.9)	1235.0 (1134.6-1480.7)	1.288	1.036-1.600	0.022*
Pre-PTPE FLR volume (mL)	355.3 (277.0-420.3)	493.6 (398.2-543.9)	3.265	1.661-6.417	0.001*
Pre-PTPE FLR ratio (%)	34.8 (28.4-37.2)	37.8 (35.4-41.7)	1.161	1.047-1.287	0.005*

Multivariable analysis

In the multivariable logistic regression model incorporating pre-PTPE FLR volume and TTR (n = 59), both variables were identified as independent predictors of insufficient hypertrophy: pre-PTPE FLR volume (OR = 4.277, 95% CI: 1.873-9.767, per 100mL increase; p = 0.001) and TTR (OR = 0.874, 95% CI: 0.770-0.993; p = 0.039) (Table 4). The model demonstrated good fit (Nagelkerke R² = 0.475; Hosmer-Lemeshow p = 0.391).

**Table 4:** Multivariable logistic regression analysis Outcome: insufficient hypertrophy (hypertrophy ratio < 1.3); n = 59. Model includes pre-PTPE FLR volume and TTR as covariates. OR for pre-PTPE FLR volume is per 100 mL increase; OR for TTR is per 1 mg/dL increase. Mathematical interdependency among FLR volume, FLR ratio, and TLV was accounted for by including only FLR volume. Collinearity between TTR and cholinesterase (Spearman's ρ = 0.558) was addressed by retaining TTR. An asterisk (*) indicates statistical significance (p < 0.05). Abbreviations: OR, odds ratio; CI, confidence interval; TTR, transthyretin; FLR, future liver remnant; PTPE, percutaneous transhepatic portal vein embolization.

Variable	OR	95% CI	p-value
pre-PTPE FLR volume (mL)	4.277	1.873-9.767	0.001*
Transthyretin (TTR) (mg/dL)	0.874	0.770-0.993	0.039*
Overall model: Nagelkerke R² = 0.475, Hosmer–Lemeshow p = 0.391 (n = 59)			

ROC analysis

**Figure 2:**
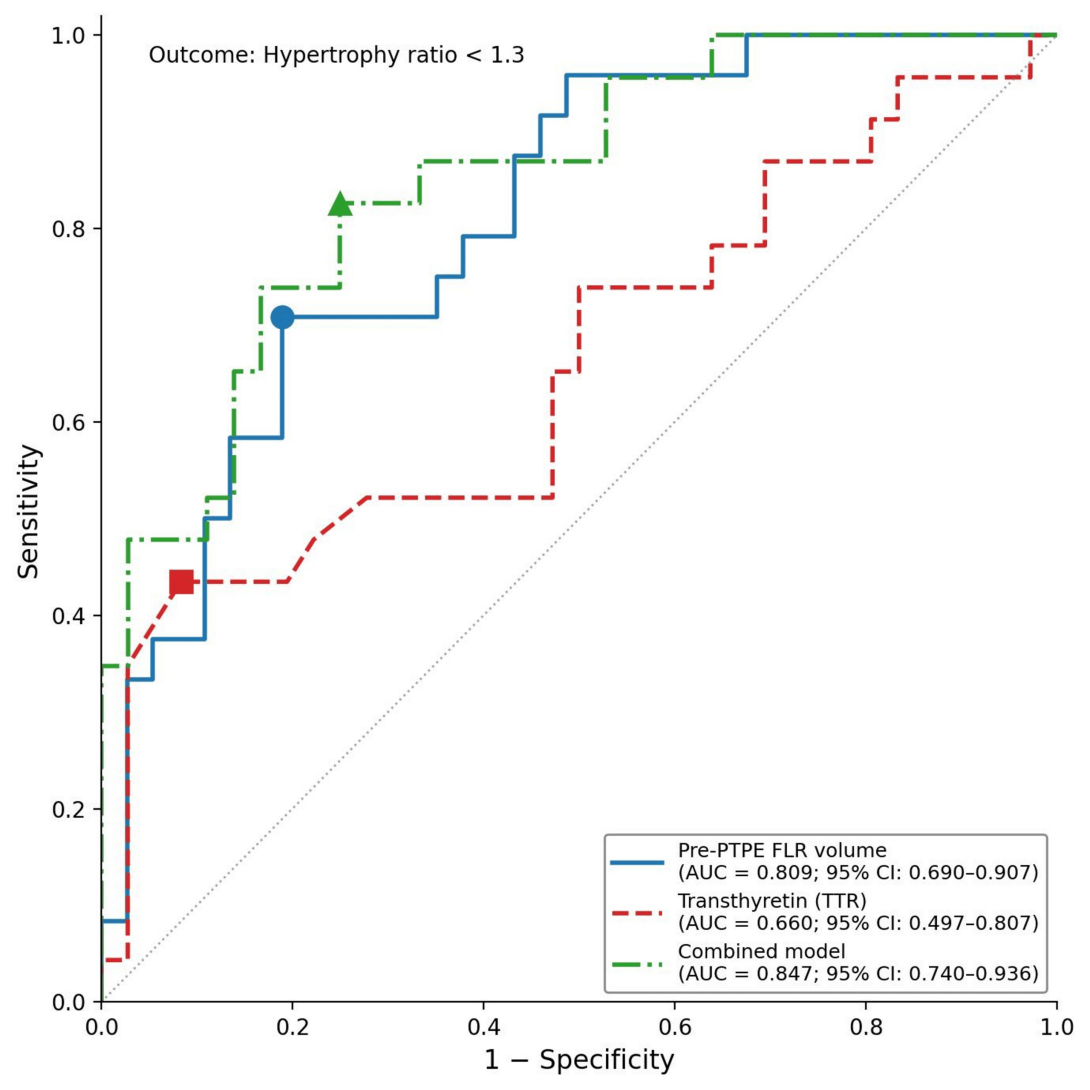
Receiver operating characteristic (ROC) curves for pre-PTPE future liver remnant volume, transthyretin, and their combination in predicting insufficient future liver remnant hypertrophy ROC curves were constructed using insufficient hypertrophy (hypertrophy ratio < 1.3) as the binary outcome (n = 59). The solid blue line represents pre-PTPE FLR volume (AUC = 0.809; 95% CI: 0.690-0.907), with an optimal cutoff of ≥ 428.3 mL (sensitivity 70.8%, specificity 81.1%), indicated by the filled circle (●). The dashed red line represents transthyretin (TTR; AUC = 0.660; 95% CI: 0.497–0.807), with an optimal cutoff of ≤ 14.9 mg/dL (sensitivity 43.5%, specificity 91.7%), indicated by the filled square (■). The dash-dot green line represents the combined model incorporating both pre-PTPE FLR volume and TTR (AUC = 0.847; 95% CI: 0.740-0.936), with an optimal point (sensitivity 82.6%, specificity 75.0%), indicated by the filled triangle (▲). Optimal cutoff values were determined by the Youden index. The diagonal dotted line represents the line of no discrimination. Abbreviations: AUC, area under the curve; CI, confidence interval; FLR, future liver remnant; TTR, transthyretin; PTPE, percutaneous transhepatic portal vein embolization.

ROC analysis was performed using insufficient hypertrophy (hypertrophy ratio < 1.3) as the binary outcome (Figure 2 and Table 5). The AUC for pre-PTPE FLR volume was 0.809 (95% CI: 0.690-0.907), with an optimal cutoff of 428.3 mL by the Youden index (sensitivity 70.8% and specificity 81.1%). The AUC for TTR was 0.660 (95% CI: 0.497-0.807), with an optimal cutoff of 14.9 mg/dL (sensitivity 43.5% and specificity 91.7%). The combined model incorporating both predictors yielded an AUC of 0.847 (95% CI: 0.740-0.936). Internal validation by LOOCV yielded AUCs of 0.804, 0.601, and 0.810 for pre-PTPE FLR volume, TTR, and the combined model, respectively.

**Table 5:** ROC analysis Outcome: insufficient FLR hypertrophy, defined as a hypertrophy ratio (post-PTPE FLR volume / pre-PTPE FLR volume) < 1.3. Optimal cutoff was defined as the point maximizing the Youden index. The 95% confidence interval for AUC was calculated by the bootstrap method (2,000 iterations). Two patients with missing TTR values were excluded from TTR ROC analysis and the combined model (n = 59). The combined model AUC was derived from predicted probabilities of the multivariable logistic regression model incorporating both pre-PTPE FLR volume and TTR. No single optimal cutoff is applicable to the combined model. Abbreviations: ROC, receiver operating characteristic; AUC, area under the curve; CI, confidence interval; TTR, transthyretin; FLR, future liver remnant; PTPE, percutaneous transhepatic portal vein embolization.

Variable	n	Insufficient hypertrophy (n)	Sufficient hypertrophy (n)	AUC	95% CI	Optimal cutoff (Youden index)	Sensitivity	Specificity
Pre-PTPE FLR volume (mL)	61	24	37	0.809	0.690 - 0.907	≥ 428.3 mL	70.8%	81.1%
Transthyretin (TTR) (mg/dL)	59	23	36	0.660	0.497 - 0.807	≤ 14.9 mg/dL	43.5%	91.7%
Combined model (pre-PTPE FLR volume + TTR)	59	23	36	0.847	0.740 - 0.936	-	-	-

Risk stratification

**Figure 3:**
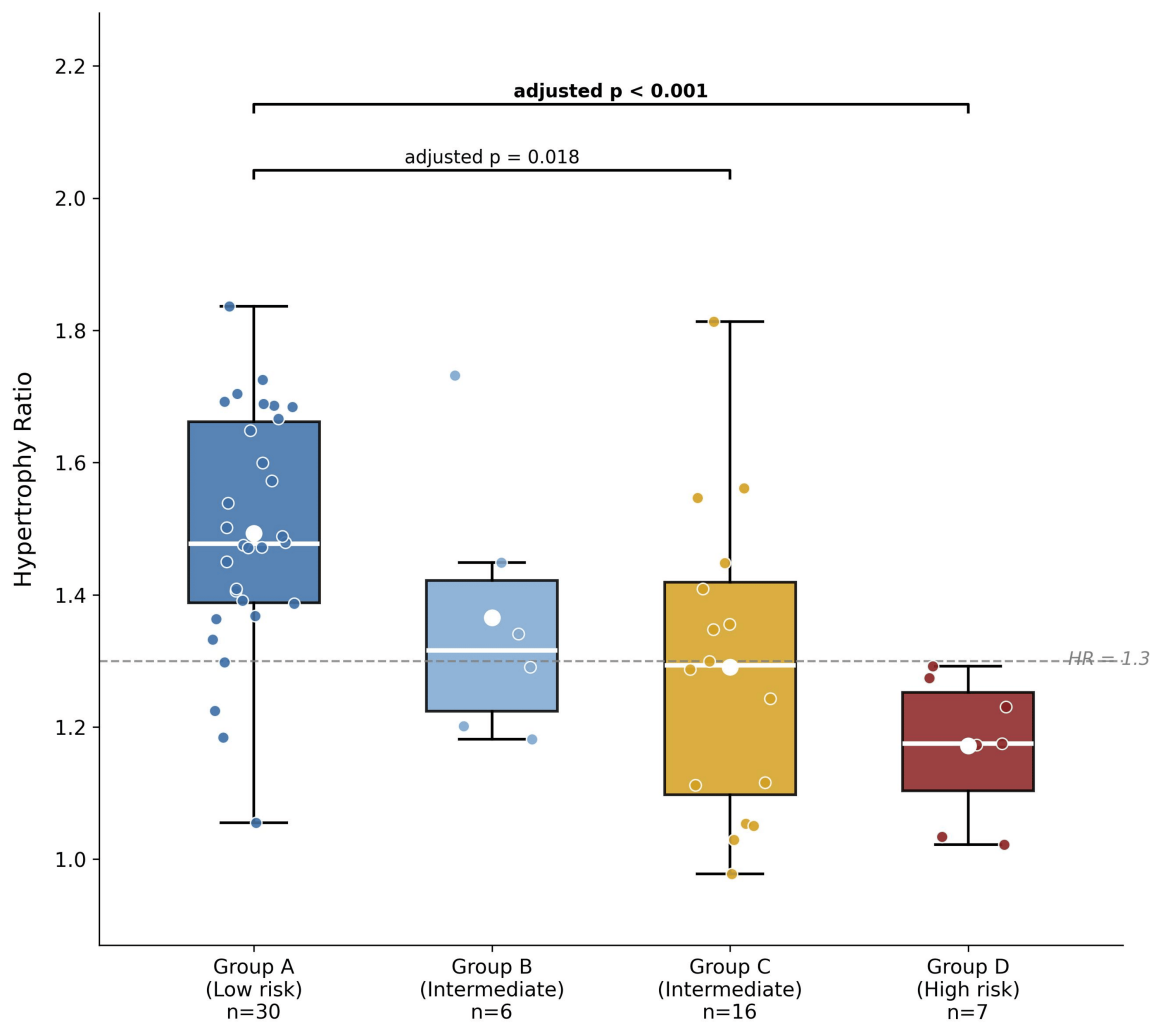
Hypertrophy ratio according to risk group stratification based on pre-PTPE future liver remnant volume and TTR cutoff values Patients (n = 59) were stratified into four groups based on the optimal cutoff values identified by receiver operating characteristic curve analysis: pre-PTPE FLR volume ≥ 428.3 mL and TTR ≤ 14.9 mg/dL. Group A (low risk, n = 30): neither risk factor present; Group B (intermediate, n = 6): TTR ≤ 14.9 mg/dL only; Group C (intermediate, n = 16): pre-PTPE FLR volume ≥ 428.3 mL only; Group D (high risk, n = 7): both risk factors present. The horizontal dashed line indicates the hypertrophy ratio threshold of 1.3. Open circles represent individual data points. The white horizontal line within each box indicates the median. Boxes represent the interquartile range, and whiskers extend to the minimum and maximum values excluding outliers. Hypertrophy ratios differed significantly across groups (Kruskal-Wallis test, p = 0.0003). Post-hoc pairwise comparisons were performed using the Mann-Whitney U test with Bonferroni correction for multiple comparisons; adjusted p-values are displayed for statistically significant pairs only. Abbreviations: PTPE, percutaneous transhepatic portal vein embolization; FLR, future liver remnant; TTR, transthyretin.

All patients (n = 59) were stratified into four groups based on the cutoff values identified by ROC analysis for pre-PTPE FLR volume (428.3 mL) and TTR (14.9 mg/dL) (Figure 3). In Group D (high-risk group, n = 7), defined by both risk factors being present, all patients experienced insufficient hypertrophy (rate: 100%). In contrast, Group A (low-risk group, n = 30), in which neither risk factor was present, had an insufficient hypertrophy rate of only 13.3%. The hypertrophy ratio differed significantly across the four groups by the Kruskal-Wallis test (p = 0.0003). Post-hoc pairwise comparisons with Bonferroni correction identified significant differences between Group A and Group C (adjusted p = 0.018) and between Group A and Group D (adjusted p < 0.001). No significant differences were observed in the remaining pairwise comparisons after correction.

Sensitivity analysis

In the sensitivity analysis using ΔsFLR as an alternative outcome measure, Spearman correlation analysis was performed in 61 patients for most variables and 59 patients for TTR due to missing values in two patients. Multivariable linear regression analysis was performed in 59 patients. On Spearman correlation analysis, both pre-PTPE FLR volume (r = -0.260, p = 0.043) and TTR (r = 0.299, p = 0.021) demonstrated significant correlations with ΔsFLR (Table 6). On multivariable linear regression analysis incorporating pre-PTPE FLR volume and TTR as covariates, both variables retained significant independent associations with ΔsFLR: pre-PTPE FLR volume (β = -0.005, 95% CI: -0.010 to -0.001, p = 0.029) and TTR (β = 0.129, 95% CI: 0.008-0.250, p = 0.037) (Table 7).

**Table 6:** Sensitivity analysis - Spearman correlation between preoperative variables and ΔsFLR (%). Spearman's rank correlation coefficients (r) and p-values are shown. An asterisk (*) indicates statistical significance (p < 0.05). n = 61 for all variables except TTR; n = 59 for TTR due to missing preoperative values in two patients. ΔsFLR (%) = (post-PTPE FLR volume - pre-PTPE FLR volume) / standard liver volume × 100; standard liver volume was estimated using the Vauthey formula (SLV = 794.41 + 1267.28 × BSA). Abbreviations: AST, aspartate aminotransferase; ALT, alanine aminotransferase; ALP, alkaline phosphatase; TTR, transthyretin; CONUT, Controlling Nutritional Status; ICG-R15, indocyanine green retention rate at 15 minutes; HH15 and LHL15, ⁹⁹ᵐTc-GSA scintigraphic indices; TLV, total liver volume; SLV, standard liver volume; FLR, future liver remnant; PTPE, percutaneous transhepatic portal vein embolization.

Variable	ΔsFLR (%)
Demographics and Comorbidities	
Age (years)	r = -0.114, p = 0.383
Diabetes mellitus	r = -0.002, p = 0.987
Viral hepatitis	r = 0.038, p = 0.771
Preoperative chemotherapy	r = -0.027, p = 0.838
Antithrombotic therapy	r = 0.065, p = 0.617
Preoperative Laboratory Values	
Total bilirubin (mg/dL)	r = -0.102, p = 0.439
AST (U/L)	r = 0.012, p = 0.929
ALT (U/L)	r = 0.145, p = 0.268
AST/ALT ratio	r = -0.162, p = 0.217
ALP (U/L)	r = -0.007, p = 0.961
Albumin (g/dL)	r = 0.159, p = 0.225
Prothrombin time (%)	r = 0.045, p = 0.735
Antithrombin III (%)	r = 0.271, p = 0.043*
Platelets (×10³/μL)	r = 0.227, p = 0.081
Transthyretin (TTR) (mg/dL)	r = 0.299, p = 0.021*
Cholinesterase (U/L)	r = 0.246, p = 0.058
CONUT score	r = -0.105, p = 0.425
ICG-R15 (%)	r = -0.198, p = 0.137
Hepatic Scintigraphy	
HH15 (×10⁻²)	r = -0.171, p = 0.188
LHL15 (×10⁻²)	r = 0.073, p = 0.578
Liver Volume Parameters	
Total liver volume, TLV (mL)	r = -0.096, p = 0.464
Pre-PTPE FLR volume (mL)	r = -0.260, p = 0.043*
Pre-PTPE FLR ratio (%)	r = -0.272, p = 0.034*

**Table 7:** Sensitivity analysis - multivariable linear regression analysis for ΔsFLR (%) Covariates: pre-PTPE FLR volume and TTR; n = 59. β, unstandardized regression coefficient. An asterisk (*) indicates statistical significance (p < 0.05). ΔsFLR (%) = (post-PTPE FLR volume - pre-PTPE FLR volume) / standard liver volume × 100. Abbreviations: TTR, transthyretin; FLR, future liver remnant; PTPE, percutaneous transhepatic portal vein embolization.

Variable	β (95% CI)	p-value
Pre-PTPE FLR volume (mL)	-0.005 (-0.010, -0.001)	0.029*
Transthyretin (TTR) (mg/dL)	0.129 (0.008, 0.250)	0.037*
Overall model fit - ΔsFLR (%): n = 59, R² = 0.165, Adjusted R² = 0.135, F p = 0.007		

To examine independence from systemic inflammation, TTR showed stronger correlations with nutritional indices (albumin: r = 0.547, p < 0.001; CONUT score: r = -0.400, p = 0.002) than with inflammatory markers (CRP: r = -0.402, p = 0.002; NLR: r = -0.272, p = 0.037) on Spearman correlation analysis (n = 59). When CRP or NLR was added as a covariate, TTR retained significance (CRP-adjusted: OR = 0.856, p = 0.032; NLR-adjusted: OR = 0.874, p = 0.046), whereas neither CRP nor NLR reached significance.

## Discussion

In this study, two independent preoperative predictors of insufficient FLR hypertrophy following PTPE were identified: pre-PTPE FLR volume and TTR. The former demonstrated a stronger association and good discriminatory performance on ROC analysis. TTR also demonstrated a significant association on both univariable and multivariable analysis; however, its discriminatory performance as a standalone predictor was modest (AUC 0.660; 95% CI, 0.497-0.807; sensitivity 43.5%), and it should be regarded as a complementary and exploratory variable rather than an independent screening tool. These findings suggest that a combination of preoperative CT volumetry and routine laboratory testing may enable preoperative risk stratification for insufficient FLR hypertrophy following PTPE.

The finding that pre-PTPE FLR volume was the strongest predictor of FLR hypertrophy after PTPE is consistent with previous reports [10,11,18]. The biological rationale underlying this association is that a smaller pre-procedural FLR volume results in a greater redistribution of portal blood flow to the non-embolized lobe, which in turn delivers stronger hepatotrophic signals, including hepatocyte growth factor (HGF), transforming growth factor-α (TGF-α), and epidermal growth factor (EGF), thereby inducing a more robust regenerative stimulus [19,20]. Supporting this mechanism, Goto et al. [21] demonstrated that the magnitude of increase in portal blood flow velocity after PTPE correlated significantly with the degree of hypertrophy. Of note, because pre-PTPE FLR volume is mathematically embedded in the denominator of the hypertrophy ratio, the observed association may partly reflect mathematical coupling. In a sensitivity analysis using ΔsFLR - the difference in standardized FLR before and after PTPE, which is not subject to this dependency - pre-PTPE FLR volume retained a significant independent association (p = 0.029), suggesting that the association is not solely attributable to mathematical coupling. 

While ICG-R15 has been reported as a predictor of FLR hypertrophy after PVE in previous studies [9,22], no significant association was observed in the present study. The median ICG-R15 in this cohort was relatively low at 8.0%, suggesting that patients with severely impaired hepatic function were underrepresented and that the distribution was narrowly concentrated, which may have limited the discriminatory power of this variable. In contrast, HH15 demonstrated a significant association with insufficient FLR hypertrophy on univariable analysis (p = 0.027), despite the overall well-preserved liver function in this cohort. This discrepancy may be mechanistically explained as follows: ICG-R15 reflects macroscopic hepatic clearance capacity, which may be highly compensated in non-cirrhotic livers, potentially masking subtle functional impairment. In contrast, HH15 reflects functional hepatocyte mass via asialoglycoprotein receptor-mediated uptake of ⁹⁹ᵐTc-GSA, potentially enabling more sensitive detection of early structural changes before they become apparent in macroscopic indices. It should be noted, however, that HH15 may also be influenced by minor fluctuations in cardiac output and systemic hemodynamics, which represents a potential limitation of this index.

Although sarcopenia and related nutritional and body composition parameters have emerged as predictors of insufficient FLR hypertrophy after PTPE [23], their assessment requires specialized software and complex calculations for muscle mass measurement. In contrast, blood-based rapid-turnover proteins may offer a simpler and more dynamic evaluation of subtle metabolic reserves even in patients without overt sarcopenia, with the additional practical advantage of being readily obtainable from routine blood tests. TTR, with a serum half-life of approximately two days - markedly shorter than that of albumin (approximately 20 days) or CHE (approximately 10 days) - is a sensitive marker reflecting short-term nutritional status, inflammation, and hepatic synthetic function in the preoperative period [24,25]. Although CHE also reached univariable significance, TTR demonstrated a stronger independent association on multivariable analysis, consistent with a prior report by Watanabe et al. [8], identifying CHE as a component of a predictive scoring system.

Although evidence regarding the clinical utility of serum TTR in predicting post-PTPE FLR hypertrophy remains limited, the present study identified TTR as an independent predictor alongside pre-PTPE FLR volume. This association was further supported by a sensitivity analysis using ΔsFLR as an alternative outcome measure, in which TTR retained a significant independent association, indicating robustness across outcome definitions. As TTR retained significance after adjustment for CRP and NLR, its predictive value may reflect hepatic synthetic reserve and nutritional status rather than systemic inflammation. This is biologically consistent with the hypothesis that higher preoperative TTR levels reflect better-preserved hepatic synthetic reserve, potentially supporting hepatotrophic signaling and a greater hypertrophic response to PTPE. It should be noted, however, that TTR demonstrated modest discriminatory performance as a standalone predictor (AUC 0.660; 95% CI: 0.497-0.807; sensitivity 43.5%), and should be regarded as a complementary variable rather than an independent screening tool. The combination of pre-PTPE FLR volume and TTR yielded an improved AUC of 0.847 (95% CI: 0.740-0.936), supporting the clinical utility of the combined model.

The exploratorily derived cutoff values for pre-PTPE FLR volume and TTR may enable preoperative risk stratification for insufficient FLR hypertrophy. In the high-risk group (Group D), in which both risk factors were met, all patients (100%) experienced insufficient hypertrophy in the present cohort, although this group comprised only seven patients, and prospective confirmation is required. In such patients, alternative hypertrophy-inducing strategies may be worth considering, as achieving the target FLR volume with PTPE alone may be challenging. Recent reports have demonstrated that liver venous deprivation (LVD), which combines PTPE with hepatic vein embolization, induces greater FLR hypertrophy than PTPE alone [26-28], representing a promising option for such high-risk patients. Preoperative risk stratification using these predictors may support individualized decision-making regarding the timing of post-PTPE volumetric follow-up and the potential need for additional interventions.

Portal vein recanalization after PTPE is an established major cause of insufficient FLR hypertrophy [18,29]. The exclusion of patients with confirmed recanalization was intended to establish a uniform baseline of complete portal occlusion, thereby isolating the biological and metabolic influences of preoperative markers such as TTR from mechanical confounding factors. However, as the study population represents only those who achieved sustained portal occlusion, the overall incidence of insufficient hypertrophy in an unselected real-world population may be higher than that observed in the present cohort. Although gelatin sponge carries an inherent risk of recanalization as an absorbable embolic agent, several technical measures were employed to minimize this risk, including selective embolization at the subsegmental level using a microcatheter and additional coil placement at the secondary branch origin when residual portal flow was identified. The median hypertrophy ratio in the entire cohort undergoing right-sided PTPE, including recanalization cases (n = 69), was 1.363 (IQR: 1.217-1.516), which was comparable to previously reported values using ethanol-based embolization (1.336) [30], suggesting that gelatin sponge-based PTPE holds the potential to achieve adequate hypertrophy when combined with meticulous subsegmental technique.

This study has several limitations. First, the single-center retrospective design and relatively small sample size limit the statistical power and generalizability of the findings. Missing TTR values in two patients represent a potential source of bias. Second, the cutoff values for pre-PTPE FLR volume and TTR were exploratorily derived from the same cohort in which their predictive performance was evaluated, potentially introducing optimism bias. Internal validation by LOOCV yielded AUCs of 0.804, 0.601, and 0.810 for pre-PTPE FLR volume, TTR, and the combined model, respectively, which were slightly lower than the corresponding bootstrap AUCs, reflecting the degree of optimism. These cutoff values should therefore be regarded as exploratory thresholds, rather than definitive clinical values. Third, all volumetric measurements were performed by a single observer, without formal intra-observer variability assessment, which may limit reproducibility. External validation in larger, multicenter cohorts is warranted. Furthermore, as gelatin sponge is an absorbable embolic agent, subclinical or partial restoration of portal flow below the threshold of imaging detection cannot be entirely excluded and may have contributed to insufficient hypertrophy in a subset of patients.

## Conclusions

In this study, pre-PTPE FLR volume was identified as the strongest preoperative predictor of insufficient FLR hypertrophy following PTPE, and TTR demonstrated a significant independent association as a complementary predictor. The discriminatory performance of TTR as a standalone predictor was modest, and it should be regarded as a complementary and exploratory variable rather than an independent screening tool. The combination of these two variables, both obtainable from routine preoperative workup, may facilitate preoperative risk stratification for insufficient FLR hypertrophy following PTPE. Given the exploratory nature and limited sample size of this study, external validation in larger, multicenter cohorts is warranted.
